# Selective benefit of the sucrose TonB-dependent receptor, SucA, in *Caulobacter crescentus*

**DOI:** 10.17912/micropub.biology.001457

**Published:** 2025-02-28

**Authors:** Erin NewRingeisen, Jacy Jordahl, Lisa Bowers

**Affiliations:** 1 St. Olaf College, Northfield, Minnesota, United States

## Abstract

Gram-negative bacteria have outer membrane proteins called TonB-dependent receptors (TBDRs) that facilitate energy-dependent transport of substrates. *Caulobacter crescentus* is a gram-negative bacterium with a large set of TBDRs, yet the function of many of these TBDRs remains uncharacterized. This study focuses on SucA, a TBDR that transports sucrose. Previous studies showed that *sucA* expression was induced in the presence of sucrose, yet did not provide a measurable fitness advantage under the conditions tested. This work identifies conditions where *sucA* does confer a significant growth advantage and provides evidence that SucA activity relies on the proton motive force, a feature of canonical TBDRs.

**Figure 1. SucA provides a growth advantage for cells exposed to an intermediate concentration of sucrose as the sole carbon source:**
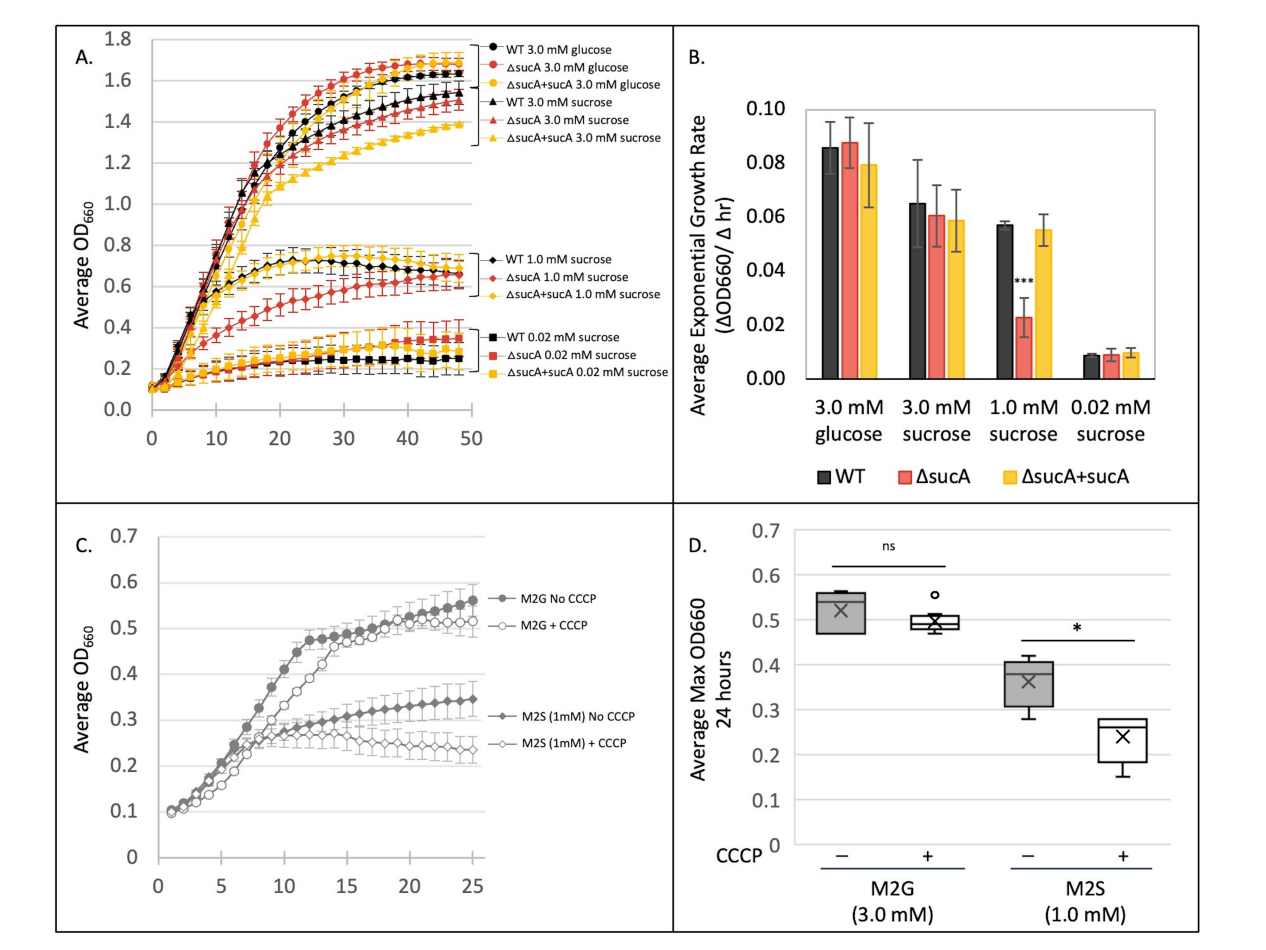
**A.** Representative growth curve measuring OD _660_ over a 48-hour period. The curves represent the average of 5 technical replicates. Error bars represent the standard error of the mean. **B.** The exponential phase growth rate of the *ΔsucA* strain is significantly less than either the wild-type or the complemented *sucA* knockout in minimal medium supplemented with 1.0 mM sucrose. The average exponential growth rate was calculated from three independent biological experiments, each with 5 technical replicates. Error bars represent the standard error of the mean. (***p-value<0.001). **C.** SucA activity is inhibited by CCCP (a non-specific proton ionophore). WT *Caulobacter* (NA1000) cells were cultured in M2 minimal medium containing 3.0 mM glucose, 3.0 mM sucrose, or 1.0 mM sucrose in the presence or absence of 6.25 µM CCCP. OD _660_ measurements were recorded every hour for 24 hours. Data represents the average of 4 technical replicates. Error bars represent the standard error of the mean. (D) OD _660_ at 24 hours. Average of three independent biological experiments each with four technical replicates. (*p-value <0.05.)

## Description

*Caulobacter crescentus* is a free-living, oligotrophic, gram-negative bacterium commonly found in soil and aquatic environments (Curtis and Brun, 2010; Wilhelm, 2018) . Like most free-living oligotrophic bacteria, it likely encounters a variety of nutrients that are scarce or fleeting in its natural environment (Hentchel et al., 2019) . To respond to changing nutrient availability, gram-negative bacteria have evolved different mechanisms for nutrient uptake across the outer membrane (OM). Outer membrane porins (OMPs) allow the passive diffusion of solutes with molecular mass < ~600 Da but for substrates that are too large or too scarce to diffuse through OMPs, *Caulobacter* relies on TonB-dependent receptors (TBDRs) for their active uptake (Noinaj et al., 2010) .

TBDRs are located in the OM of gram-negative bacteria and bind substrates with high affinity. Substrate binding causes a conformational change, allowing the TBDR to interact with TonB, a protein that bridges the periplasmic space (Schauer et al., 2008). TonB physically couples the TBDR with a cytoplasmic membrane (CM) motor protein complex (ExbB-ExbD), which is powered by the proton motive force (pmf) (Wiener, 2005; Noinaj et al., 2010; Silale and Van Den Berg, 2023) . This interaction uses energy derived from the pmf to drive transport of the substrate across the OM. Different TBDRs have been shown to transport a wide variety of substrates including vitamin B _12_ complex, iron chelators, transition metals, carbohydrates, and aromatic compounds (Menikpurage et al., 2019; Mazzon et al., 2014; Neugebauer et al., 2005; Blanvillain et al., 2007; Eisenbeis et al., 2008; Lohmiller et al., 2008; Modrak et al., 2018; Balhesteros et al., 2017; Presley et al., 2014; Fujita et al., 2019). While most gram-negative bacteria encode a few TBDR genes (Blanvillain et al., 2007) , *Caulobacter crescentus* is predicted to encode 67 different TBDRs (Nierman et al., 2001) , most of which are yet to be characterized. Furthermore, many of these TBDRs are conserved throughout the α-proteobacteria, which suggests they may confer a selective benefit in some environments, yet they are often dispensable for growth under typical laboratory growth conditions (Blanvillain et al., 2007; Christen et al., 2011; Déjean et al., 2013) .

*SucA provides a selective benefit to cells growing with sucrose as the sole carbon source.*

SucA is a *Caulobacter* TBDR that transports sucrose. Previous studies determined its expression is strongly induced in the presence of sucrose, yet it does not confer a growth advantage for cells under typical laboratory growth conditions when sucrose is supplied at 3.0 mM in the growth media (Modrak et al., 2018) . We wondered if SucA might confer a growth advantage at a lower concentration of sucrose, since natural aquatic environments have been reported to have sucrose concentrations in the µM range (Sogin et al., 2022) . To test this, we compared the growth rate of three isogenic strains: WT *Caulobacter* (NA1000), an in-frame unmarked *sucA* deletion strain ( *ΔsucA* ), and the complemented deletion strain with *sucA* expressed from its native promoter on a low-copy plasmid ( *ΔsucA+pSucA* ) . The growth of these three strains was measured over 48 hours in minimal media containing either glucose (3.0 mM) or sucrose (0.02 mM, 1.0 mM or 3.0 mM) as the sole carbon source ( Figure 1A ). Maximum cell density depends on carbon content and may not reveal subtleties in cell division rates; therefore, we analyzed fitness of the three strains by examining the exponential growth rate ( Figure 1B ). As expected, all three strains had equal exponential growth rates when glucose (3.0 mM) was the sole carbon source and at the highest concentration of sucrose (3.0 mM) where sucrose would be expected to enter the cells via facilitated diffusion. At the lowest concentration of sucrose tested (0.02 mM), there was not significant growth for any of the three strains in 48 hours (with this low amount of carbon, cells could at most perform one doubling). However, at the intermediate sucrose concentration (1.0 mM), the cells expressing *sucA* (WT and *ΔsucA* + sucA) had a significantly greater exponential growth rate compared to cells lacking *sucA* ( *ΔsucA* ) ( Figure 1A and 1B). Typical TBDRs are known to have a high affinity for their substrate, often with a K _d_ in sub-µM range (Neugebauer et al., 2005; Blanvillain et al., 2007) so it was surprising to observe a significant difference in exponential growth rate between 3.0 mM sucrose and 1.0 mM sucrose. However, after 48 hours, the cells lacking *sucA* ( *ΔsucA* ) did eventually reach the same maximum optical density as the cells expressing *sucA* ( Figure 1A ). Thus, there may be other slower uptake mechanisms for sucrose.

*SucA activity relies on the proton motive force.*

A canonical feature of TBDRs is their reliance on the pmf generated across the CM to drive the transport of nutrients across the OM (Bradbeer and Woodrow, 1976) . The proton gradient can be disrupted by the non-specific proton ionophore carbonyl cyanide-m-chlorophenylhydrazone (CCCP). In order to test whether SucA-mediated transport of sucrose across the OM is dependent upon the pmf of an intact CM, growth of WT *Caulobacter* cells was monitored in the presence and absence of a non-lethal concentration of CCCP. If SucA activity depends on the pmf, its activity should be reduced in the presence of CCCP and this effect would be most obvious at lower concentrations of the substrate. WT *Caulobacter* cultures (NA1000) containing either 3.0 mM glucose (M2G) or 1.0 mM sucrose as the sole carbon source were incubated with or without 6.25 µM CCCP and the culture density was measured for 24 hours ( Figure 1C ). For cells grown in M2G, there was no significant change in optical density after 24 hours with and without CCCP ( Figure 1C and 1D); thus, we determined that cell viability and glucose uptake was not affected at this concentration of CCCP. However, the growth of cells in M2S with 1.0 mM sucrose was significantly inhibited by the addition of CCCP ( Figure 1C and 1D). The inhibitory effect of CCCP was first observed in the M2S cultures after around 10 hours of incubation. In the M2S cultures, the cells treated with CCCP transitioned to stationary phase earlier than the cells without CCCP, and reached a lower optical density after 24 hours. Thus, CCCP diminishes the selective advantage of SucA, evidence that SucA-mediated transport depends on the energy from the pmf, typical of a canonical TBDR.

In other environmental bacteria, some TBDRs that transport carbohydrates are dispensable for growth in nutrient media but essential for growth and virulence on plant leaves (Blanvillain et al., 2007; Déjean et al., 2013) . Similarly, in studies shown here, the *Caulobacter crescenuts sucA* gene is dispensable for growth at a relatively high sucrose concentration (3.0 mM) but confers a significant growth advantage at a reduced sucrose concentration (1.0 mM). Furthermore, this growth advantage provided by the *sucA* gene depends on an intact pmf. Together, these findings contribute to a more nuanced understanding of the role and selective advantage of TBDRs and highlight the importance of laboratory growth conditions when studying the function and selective benefit of nutrient uptake systems.

## Methods

**Bacterial strains and growth conditions**

The *Caulobacter crescentus* strains in this study were NA1000 (referred to here as WT), LB235 (Modrak et al., 2018) (a non-polar *sucA* deletion strain which is referred to here as Δ *sucA* ), and LB236 (Modrak et al., 2018) (the *sucA* deletion strain complemented with *sucA* on a low copy plasmid under its own promoter, referred to here as Δ *sucA* + p *SucA* ). All strains were incubated at 30°C in M2 minimal medium (Ely, 1991) , supplemented with either 3.0 mM D-glucose (M2G), 3.0 mM sucrose (M2S), 1.0 mM sucrose, or 0.02 mM sucrose ***.*** Kanamycin was added to all cultures of LB236 (Δ *sucA* + p *SucA)* at 25 μg/ml.

**Growth Assay:** *NA1000* , Δ *sucA* , and Δ *sucA* + p *SucA* were grown in M2G overnight and then spun down and the spent media was removed. Cells were then washed in 1 mL of M2 minimal medium supplemented with either 3.0 mM D-glucose, 3.0 mM sucrose, 1.0 mM sucrose or 0.02 mM sucrose and then resuspended in 1 mL of the same media to a starting OD _660_ = 0.05.

For the samples treated with carbonyl cyanide-m-chlorophenylhydrazone, CCCP was added with a final concentration of 6.25 µM after cells were washed and resuspended. Five technical replicates for each sample were loaded into a 96-well plate with 200 μL of culture per well. The plates were incubated at 30°C with aeration and the optical density (OD _660_ ) was recorded every hour with a BioTek Epoch-2 microplate reader running Gen5 TS 2.09 software. The first derivative of the growth curve (change in OD _660_ vs time) was used to identify the period of exponential growth and exponential growth rate was calculated for each sample (ΔOD/ΔT). Growth curves were conducted in three independent biological experiments, each with five technical replicates. (*p-value <0.05)
